# Efficient field correction of low-cost particulate matter sensors using machine learning, mixed multiplicative/additive scaling and extended calibration inputs

**DOI:** 10.1038/s41598-025-02069-w

**Published:** 2025-05-27

**Authors:** Slawomir Koziel, Anna Pietrenko-Dabrowska, Marek Wojcikowski, Bogdan Pankiewicz

**Affiliations:** 1 Engineering Optimization & Modeling Center, Reykjavik University, Reykjavik, 102 Iceland; 2 Faculty of Electronics, Telecommunications and Informatics, Gdansk University of Technology, Gdansk, 80-233 Poland

**Keywords:** Air pollution monitoring, Particulate matter, Low-cost sensing systems, Correction techniques, Field calibration, Time series, Neural networks, Surrogate modeling, Electrical and electronic engineering, Computational science

## Abstract

Particulate matter (PM) stands out as a highly perilous form of atmospheric pollution, posing significant risks to human health by triggering or worsening numerous heart, brain, and lung ailments, and even increasing the likelihood of cancer and premature mortality. Therefore, ensuring accurate monitoring of PM levels holds paramount significance, particularly urban zones of dense population. Still, achieving precise readings of PM concentration demands the use of bulky and costly equipment, typically stationed at widely spaced reference sites. The rise in popularity of low-cost PM sensors as potential substitutes has been noted, although their reliability is hampered by manufacturing flaws, instability, and susceptibility to environmental variations. In this work, we introduce a novel approach to field calibration for cheap PM sensors. Our method integrates multiplicative and additive corrections, with coefficients determined by an artificial neural network (ANN) surrogate. The ANN model accounts for environmental parameters and the sensor’s PM readings as inputs, with its architecture fine-tuned to ensure optimal generalization capability. Additionally, we consider an extended set of input parameters, including local temporal changes of environmental variables, and short sequences of low-sensor readings, to further enhance calibration reliability. We validate our technique using a non-stationary measurement equipment alongside reference data acquired by government-approved reference stations in Gdansk, Poland. The obtained values of coefficients of determination reach as high as 0.89 for PM_1_, 0.87 for PM_2.5_, and 0.77 for PM_10_, respectively, while the root mean square error (RMSE) is merely 3.0, 3.9, and 4.9 µg/m³. Such a performance positions the calibrated low-cost sensor as a potential alternative to stationary measurement equipment.

## Introduction

Polluted air worsens citizens’ wellbeing and increases morbidity. Some works estimate that nearly nine million premature deaths per year are caused by atmospheric contamination^1,2^. European Environmental Agency (EEA)^3^ considers polluted air in urban areas as the predominant health concern related to environmental conditionsleading to chronic diseases and increased mortality^4^. Most of the current research on PM (Particle Matter) pollutants concentrates on fine PM_2.5_ particles of diameter below 2.5 μm. This is because small-diameter particles are deemed most harmful, as, once inhaled, they can permeate the lungs at a considerable depth in comparison to larges particles^5^. Multiple reports unfailingly link PM_2.5_ with raised incidence of cardiac conditions^6,7^, cancer diseases^8,9^, and prematurely born infants^10^. In 2020, according to World Health Organization (WHO), 96% of European Union’s urban population experienced concentrations of PM_2.5_ surpassing the admissible concentration of 5 micrograms per cubic meter (µg/m3)^3^. The primary sources of PM_2.5_^11^ include household combustion, traffic-related sources, as well as mineral dust emitted at construction sites and industrial processes of increased temperature (especially steel processing)^11^.

At present, stationary government-approved reference stations are employed for precise monitoring of PM_2.5_. The most common measurement techniques are particle gravimetric methods, where the molecules are deposited on filters and then stabilized. Finally, the particle mass is quantified by accredited laboratories with the filters weighted before and after sampling. Despite being highly accurate, the said method is expensive and time-consuming. Moreover, it generates data sets of unsatisfactory spatial and temporal resolution, making it difficult to assess air PM_2.5_ concentrations in their entirety and complexity^12,13^. As a result, high-cost government facilities are predominantly used as a benchmark for calibrating data collected with the use of affordable low-cost sensors (LCS). This has instigated the development of numerous field calibration techniques. As a result, nowadays, high-cost government facilities are predominantly used as a benchmark for calibrating data collected using affordable low-cost sensors.

LCSs hold significant potential for assessing exposure to ambient pollutants with enhanced spatial density^14,15^. The benefits of using LCSs include enhancement of spatial coverage, reduced energy consumption, straightforward operation and easy maintenance, as well as relocation flexibility. LCSs may be employed independently or as supplements to existing government stations^16^. They may also be integrated into dense stationary networks^17^, deployed within vehicular networks^18,19^, or utilized as wearable instruments^20–22^ for personal observation.

Inexpensive devices predominantly utilize optical measurement techniques, which provide an approximation of mass concentrations directly measured by reference facilities. The drawbacks of optical particle sensors include decreased accuracy, inferior dependability, measurement inconsistency, but also necessity of calibration^23–26^. The basic operating principle of cost-effective commercial PM sensors is based on rapid yet imprecise light scattering^27^. Hence, significant discrepancies are observed between LCS readings and those of reference stations^28^, one of the reasons being the fact that increased relative humidity may lead to hygroscopic particle growth, resulting in dry mass overestimation^29–31^. LCSs’ inability to detect particles of diameters lower than a threshold value poses another challenge. Moreover, research conducted in laboratory-controlled settings indicates considerable accuracy variation of concentrations of pollutants across various optical sensors^32^. Thus, LCSs-acquired data needs to undergo a thorough calibration.

In recent years, a considerable rise in calibration techniques occurred. One of the least intricate approaches is linear regression, where sensor readings are utilized as the sole input^33–35^. Multivariate linear regression is slightly more complicated by including supplementary input variables (e.g., temperature or humidity) in the calibration process^36–39^. An alternative method involves gain-offset model^40–42^, which accounts for additive and multiplicative bias. Still, aforementioned straightforward techniques fail to address the nonlinearities of sensors^43^, which can be successfully handled by machine learning (ML) approaches^44–46^. The reported frameworks include random forest^47,48^, support vector regression^49,50^ or gradient boosting methods^51,52^. Recently, a growing popularity of correction techniques using neural networks (NNs) has been observed, such as Feedforward NNs^53^, Long Short-Term Memory NNs^54,55^, Recurrent NNs^56,57^, and convolutional NNs^58,59^.

This study aims to introduce a novel methodology for a reliable calibration of cheap PM sensors. Our method involves a collective multiplicative and additive sensor correction, with coefficients determined by an artificial neural network (ANN) surrogate in the form of a multi-layer perceptron (MLP). Environment-related data (temperature, humidity, and atmospheric pressure) and the current PM reading from LCS serve as inputs for calibration. A dedicated hyper-parameter controls the weight distribution between multiplicative and additive scaling, which is optimized alongside the MLP architecture during surrogate training to enhance model generalization. Augmenting correction reliability, supplementary input variables are included, incorporating temporal changes of environmental variables and short time sequences of previous sensor measurements. These additions enable the MLP surrogate to learn typical temporal dependencies between environmental parameters and PM sensor outputs, ultimately improving calibration reliability. Reference data was collected from multiple government monitoring facilities across the city of Gdansk. As demonstrated, all components of our calibration framework contribute to its exquisite performance.

## Sensor hardware and software

This section describes a custom-designed measurement platform utilizing affordable PM sensors for outdoor air pollution monitoring. The included tailored hardware and software facilitated data acquisition to compile a comprehensive dataset from the sensors. The procedure for collecting reference measurements is discussed in Sect. Collecting Reference Data. Section ML-based Sensor Correction elucidates the PM sensor calibration approach.

The primary hardware module is the Beaglebone^®^ Blue microprocessor board^60^ tailored for robotic applications. This board meets the project’s needs due to robust processing capabilities and numerous connectivity options. It is a compact device (87 × 55 mm) featuring an ARM Cortex-A8 processor equipped with 64KB RAM. The board also incorporates a circuit for charging a 2-cell Li-Po battery, enabling the platform to operate autonomously for about 24 h with a 7.4 V, 4400mAh battery. For prolonged experimentation, an external power source ranging from 9 V to 18 V DC can be connected. Long-distance communication, essential in field testing and calibration, was ensured by employing a compact modem^61^ incorporating a GPS module for geolocation purposes.

The low-cost sensor of choice was a SPS30 Sensirion device^62^ selected for its affordability, electrical characteristics, and size. At the time of equipment development, the availability of low-cost PM sensors with sufficiently high declared quality was limited, so the sensor SPS30 meeting the requirements was used. As new sensors become available, their evaluation is planned as an extension of the study as part of future work. It uses an optical method to measure PM, where a fan moves air through the laser beam, and a sensor detects PM particles. The sensor can measure PM concentrations from 0 to 1000 µg/m^3^ and offers specific accuracies for different PM sizes. The measurement system runs on Ubuntu Linux version 18.04 LTS and features two layers of software: (i) drivers implemented in C and Python for interfacing the GSM modem, along with the PM and environmental sensors, and (ii) the primary software (Python), which manages the system’s overall synchronization and operation.

**Fig. 1:**
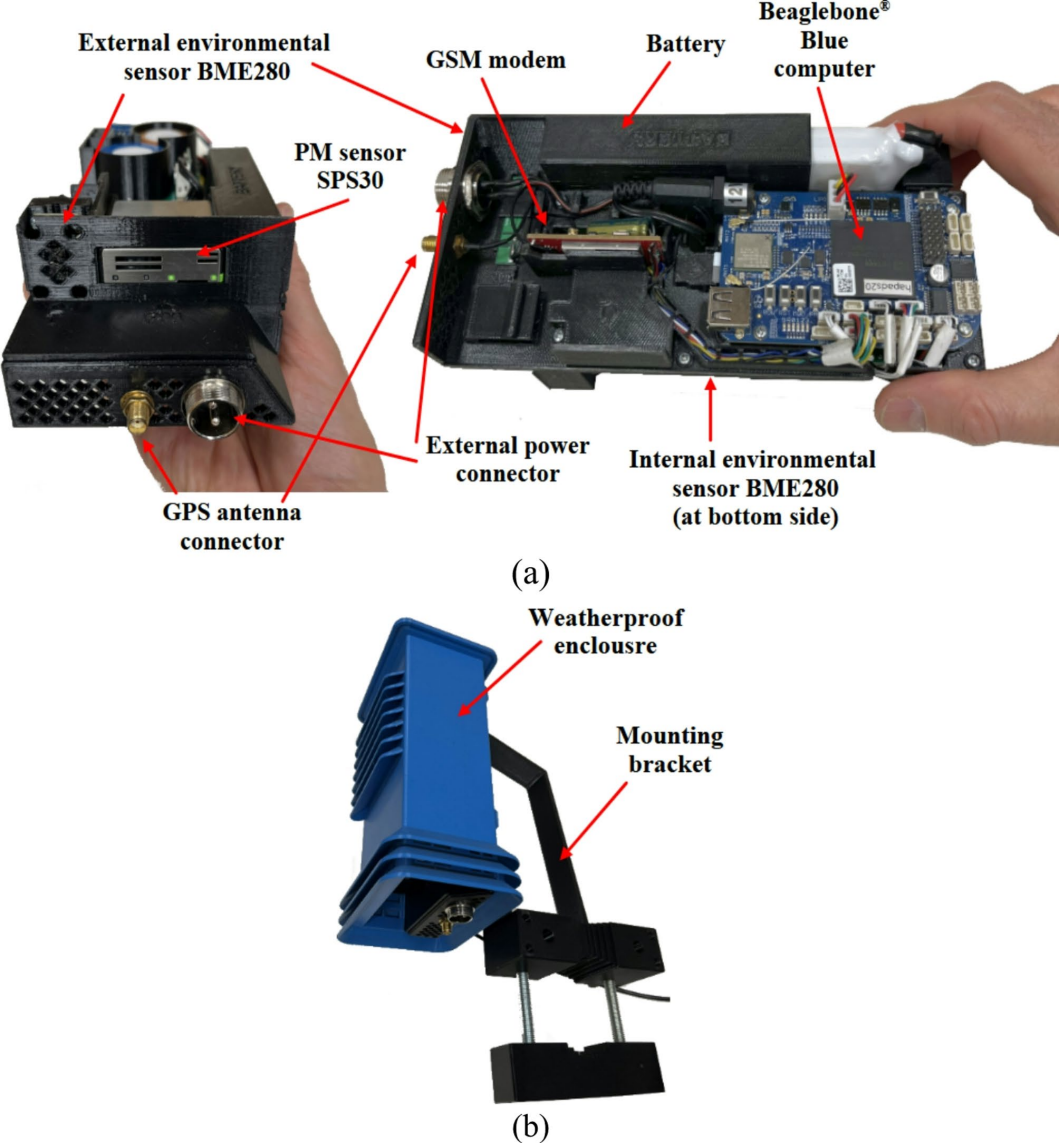
Hardware platform: (**a**) internals of the hardware, (**b**) weatherproof enclosure with the mounting bracket.

Designed for outdoor use, the platform is encased in a weather-resistant enclosure, as seen in Fig. 1. All components are mounted on a PET-G chassis, produced using 3D printing with fused deposition modeling (FDM) technology, which also was used to create the enclosure and mounting bracket. This design facilitates maintenance by providing convenient access to inner components.

## Collecting reference data

Several air quality monitoring stations were established by the municipality of Gdansk, Poland, which are handled by the ARMAG foundation (Agencja Regionalnego Monitoringu Atmosfery Gdansk-Gdynia-Sopot; English: Agency of Regional Air Quality Monitoring in Gdansk metropolitan area)^63^. The equipment operated by ARMAG serves as the official reference for the entire city area, and no alternative PM data sources are currently available. These stations are kept in air-conditioned containers, and their equipment includes professional-grade devices for automated measurement of key air pollutants: PM_1_, PM_2.5_, PM_10_, carbon monoxide, and nitrogen oxide, alongside environmental parameters (temperature, atmospheric pressure, direction and speed of wind, precipitation, and humidity). For particulate matter quantification, we employ GRIMM #180 Environmental Dust Monitors, utilizing the 90º laser light scattering technique. The measurements procured from these stations constitute the reference dataset for the calibration of cheap PM sensors. Table 1 provides the important details of the GRIMM analyzer. The measurements are taken hourly and are disseminated daily via the ARMAG foundation’s website. To amass data gathered within extended periods, a Python script was devised to automate the extraction of data from the ARMAG’s website into CSV format.

**Table 1:** Characteristics and performance metrics of the reference particulate matter analyzer: environmental GRIMM #180 dust monitor.

Parameter	Value
Measurement principle	90° light-scattering method
Measurement channels	PM_10_, PM_2.5_, and PM_1_
Mass-concentrations measurement range	0.1–1.500 µg/m^3^
Precision	± 2%
Smallest detectable particle size	0.25 μm
Light source and power	Laser diode, wavelength 685 nm P_norm_ 0.5/32 mW, P_max_ 60 mW (multiplex)
Measurement time	From 1 min until continuous
Dimensions	483 × 177 × 400 mm
Weight	15 kg

**Fig. 2:**
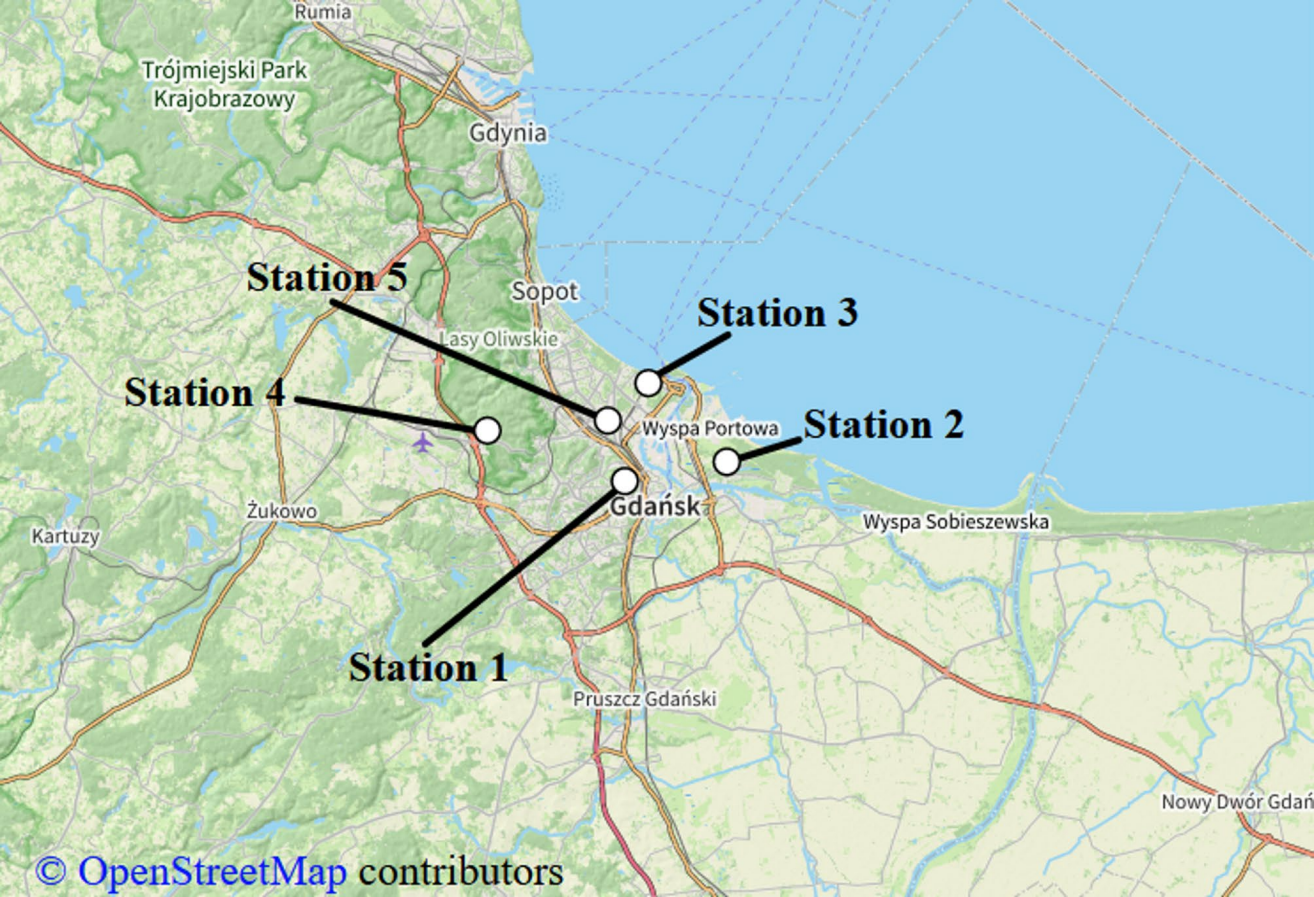
Geographic distribution of ARMAG’s air quality monitoring stations in Gdansk, Poland. Map from OpenStreetMap^64^.

The hardware units employing cheap PM sensors were placed in the proximity of the ARMAG’s reference stations (cf. Figure 2) over a period of about two months (March to May 2023). The equipment was mounted at the reference stations. The units used GSM modems to transfer measurement data to the cloud, from when the data was retrieved in CSV format containing raw sensor readings. This dataset, in conjunction with CSV file comprising reference data, constituted the foundation for the development of the correction model elaborated on in Sect. 4.

## ML-based sensor correction

This section outlines the proposed technique for LCS calibration. The section is structured as follows: Sect. Calibration Task provides a comprehensive formulation of the sensor calibration task. Joint multiplicative and additive scaling is explained in Sect. Affine Output Scaling. Section ANN Calibration Model delves into the calibration model in the form of the artificial neural network and discusses auxiliary calibration inputs. The operational flow of entire framework is detailed in Sect. Calibration Procedure.

### Calibration task

**Fig. 3:**
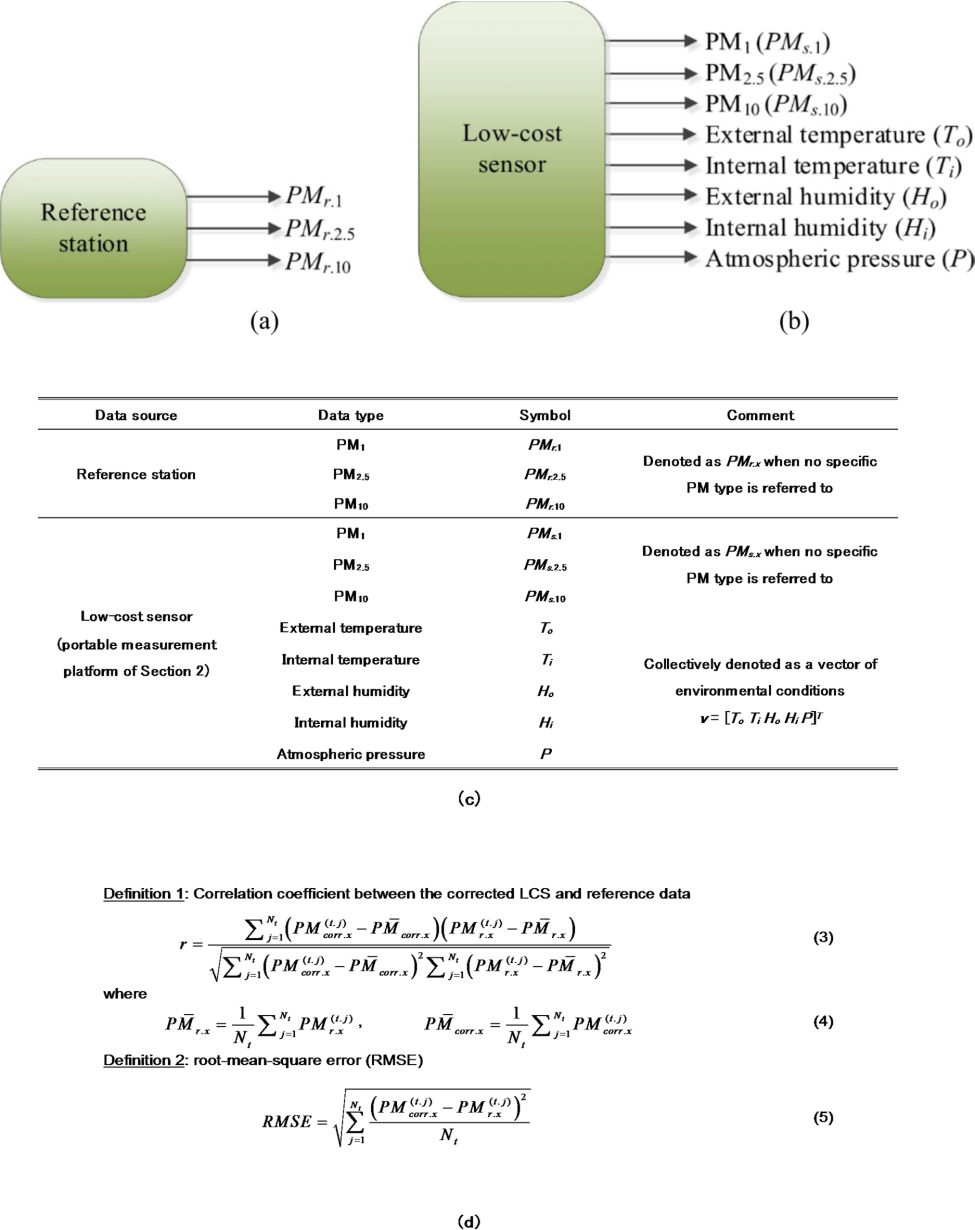
Low-cost sensor and reference station outputs: (**a**) reference PM_*x*_ readings; (**b**) LCS PM_*x*_ readings. The supplementary data includes: external temperature and humidity, *T*_*o*_, and *H*_*o*_, respectively, internal temperature and humidity, *T*_*i*_, and *H*_*i*_, respectively, as well as atmospheric pressure *P*; (**c**) utilized notation; (**d**) definitions of performance metrics: correlation coefficient, and RMSE.

The data collected by both reference stations and LCSs has been illustrated in Fig. 3. In this work, we consider particulate matter (PM) pollutions, referred to as *PM*_*r.x*_. The subscript *x* refers to a particular type of PM, i.e., 1, 2.5, or 10 (ultrafine, fine, coarse; size in µm). LCS yields also environmental parameters such as temperature, humidity, and atmospheric pressure. Distinct sensors are employed to measure external and internal conditions, reflecting variations attributable to heating from the embedded electronic devices within the hardware unit. Given the sensor’s sensitivity to temperature and humidity, both internal and external parameters are considered as calibration factors to bolster the calibration process’s dependability. The notation employed to represent the outputs of the reference stations and LCSs has been consolidated in Fig. 3(c).

We will denote as *N* the total number of collected data samples. The dataset is divided into the training and testing parts of the sizes *N*_*b*_ and *N*_*t*_ samples, respectively; *N*_*t*_ is set to be about 20% of *N*. The following notation will be employed:

- *PM*_*r.x*_^(*b.j*)^, *j* = 1, …, *N*_*b*_ – reference training samples;
- *PM*_*s.x*_^(*b.j*)^, *j* = 1, …, *N*_*b*_ – LCS training samples (PM values);
- ***v***^(*b.j*)^, *j* = 1, …, *N*_*b*_ – LCS training samples (environmental data);
- *PM*_*r.x*_^(*t.j*)^, *j* = 1, …, *N*_*t*_ – reference testing samples;
- *PM*_*s.x*_^(*t.j*)^, *j* = 1, …, *N*_*t*_ – LCS training samples (PM);
- ***v***^(*t.j*)^, *j* = 1, …, *N*_*t*_ – LCS training samples (environmental data).

The notation used for the calibration model is *C*(*PM*_*s.x*_,***v***;***p***), with ***p*** referring to the aggregated parameter vector (e.g., weights of the NN model, see Sect. ANN Calibration Model). The surrogate yields predicted output of the calibrated LCS. Identification of parameters ***p*** is carried out to better the alignment between LCS data and the reference readings. The optimal vector p^*^ of parameters of calibration model is obtained by minimizing the loss function (mean square error, MSE):

1 $${{\boldsymbol{p}}^*}=\arg \mathop {\hbox{min} }\limits_{{\boldsymbol{p}}} \frac{1}{{{N_b}}}\sum\limits_{{j=1}}^{{{N_b}}} {{{\left( {PM_{{r.x}}^{{(b.j)}} - C\left( {PM_{{s.x}}^{{(b.j)}},{\boldsymbol{v}}_{{}}^{{(b.j)}},{\boldsymbol{p}}} \right)} \right)}^2}}$$

The corrected LCS reading equals

2 $$P{M_{corr.x}}=C\left( {PM_{{s.x}}^{{}},{\boldsymbol{v}},{{\boldsymbol{p}}^*}} \right)$$

We use two performance metrics, a coefficient of determination between the corrected LCS and reference data, along with the RMSE error (cf. Figure 3(d) for definitions), which assess the closeness of sensor readings with respect to the reference data.

### Affine output scaling

The employed sensor calibration scheme involves affine response scaling with joint additive and multiplicative corrections. By additive correction, we understand a functional dependence between the uncorrected (raw) and calibrated sensor reading, which is in the form of adding a correction term to the latter. Whereas multiplicative correction is multiplying the sensor’s reading by the correction term. The additive correction is preceded by the multiplicative one. The equilibrium between these correction types is governed by an adjustable coefficient.

**Fig. 4:**
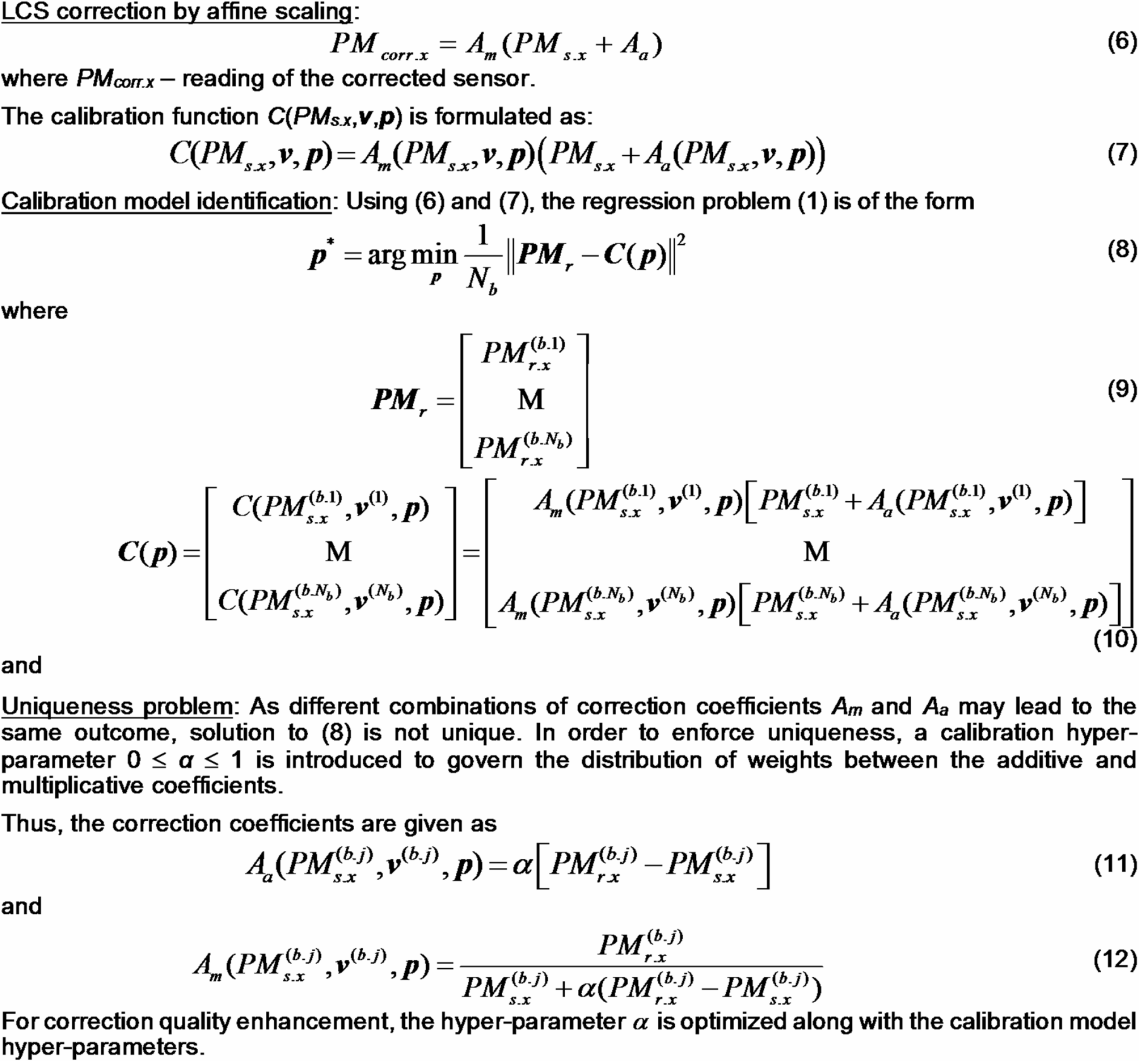
Mixed additive and multiplicative response correction of LCS.

Figure 4 summarizes the proposed correction approach. Because the LCS output PM_*s.x*_ is scalar, determination of correction coefficients (multiplicative and additive ones) is non-unique. To ensure uniqueness, we introduce hyper-parameter *α*, controlling the balance between both types of scaling. This parameter will be optimized simultaneously with identification of the neural network calibration model as elaborated on in Sect. ANN Calibration Model.

### ANN calibration model

In our work, LCS calibration utilizes artificial neural networks^65^. Specifically, we employ a feedforward ANN in the configuration of a multi-layer perceptron^66,67^. With three fully-connected hidden layers, MLP is flexible enough o while remaining resilient to overfitting—an essential characteristic given the significant dissimilarities between the reference and LCS measurements. In addition to the conventional training of network weights, we also optimize the number of neurons in the hidden layers, along with the affine scaling coefficient *α* discussed in Sect. Affine Output Scaling.

The training setup of the MLP correction model is as follows: model is trained via backpropagation Levenberg–Marquardt routine^68^ (sigmoid activation function, max. 1000 epochs, loss function in the form of mean-square error (MSE), and a random training/testing data split). The model’s inputs consist of environmental parameters (represented as vector ***v***) and the particulate matter measurement from the sensor (*PM*_*s.x*_). The model outputs correction coefficients *A*_*a*_ and *A*_*m*_.

Owing to its architectural simplicity, the network effectively mitigates the inherent noise in PM measurements. Moreover, with quick model training (completed within a few seconds), it becomes feasible to implement a nested optimization procedure. This procedure enables testing of various architectural arrangements and adjusting relevant hyper-parameters to enhance the model’s generalization capability.

**Fig. 5:**
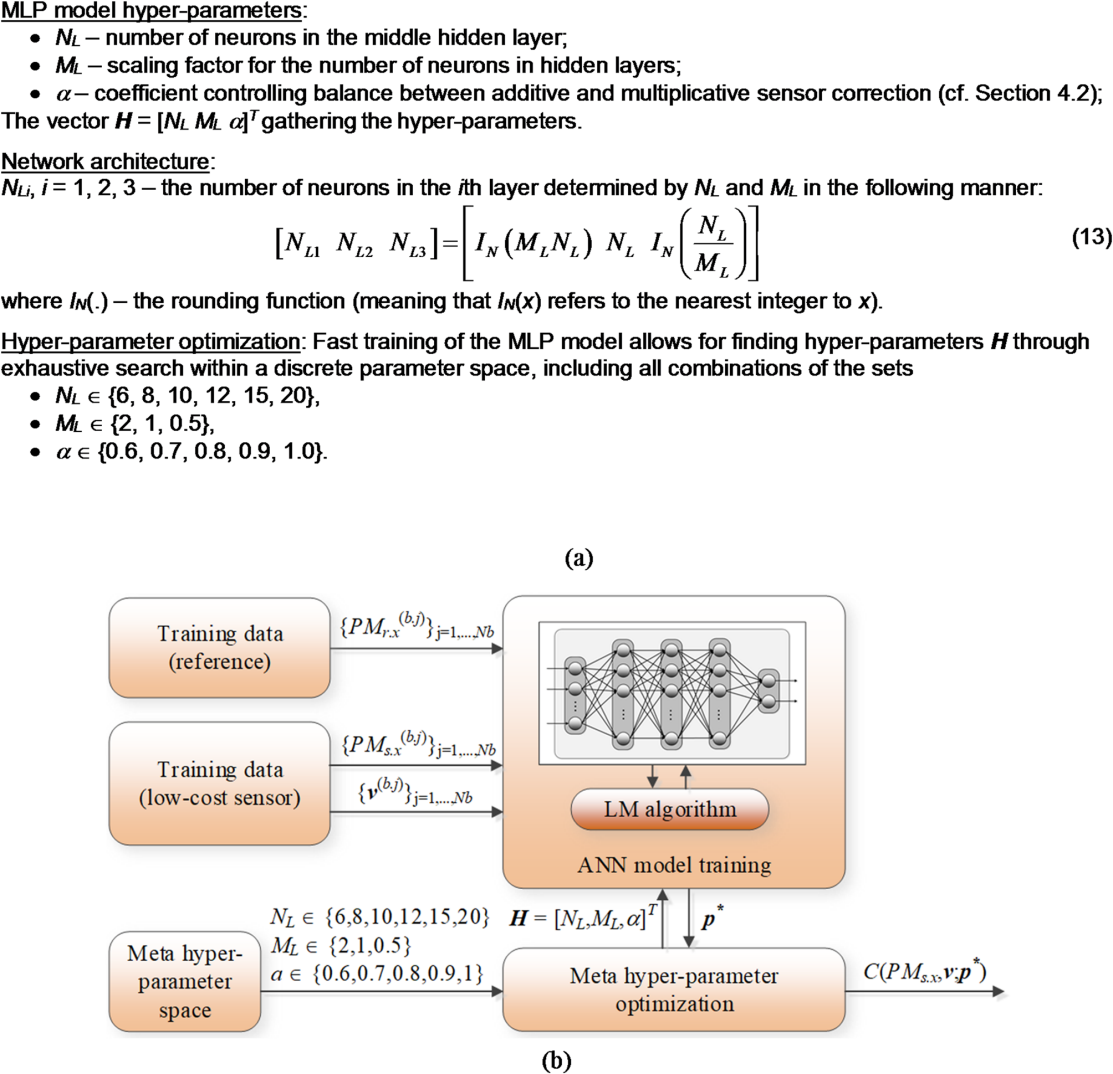
Procedure of identifying the correction model with the meta hyper-parameters ***H*** = [*N*_*L*_ *M*_*L*_ *α*]^*T*^ simultaneously adjusted (**a**) MLP model hyper-parameters and their optimization setup; (**b**) flowchart of the entire procedure. The ANN model training is carried out for each meta hyper-parameter combination to find the optimal configuration and the best value of the scaling coefficient *α*.

Figure 5 presents the calibration model’s identification process involving optimization of the model’s hyper-parameters. Notably, an exhaustive search is conducted within a discrete space of hyper-parameters, encompassing multiple combinations of ANN architectures and coefficient *α*. For each vector ***H***, the network undergoes fifty training iterations, and the best-performing model is chosen. Numerous training runs are essential due to the random (internal) partitioning of data into training and testing sample.

#### Auxiliary calibration inputs

For calibration process enhancement, the standard set of calibration model inputs, i.e., environmental variables (vector ***v***) and PM measurements collected by LCS (*PM*_*s.x*_) will also include differentials of the environmental parameters summarized in Table 2. More specifically, we will consider the following quantities Δ*x* = [*x*(0) – *x*(–*dt*)]/*dt*, with *x*(*t*) referring to a parameter measured by the sensor, whereas *dt* is the time gap between acquiring the readings (here, one hour for both the reference stations and LCS). If the collected differential vector Δ***v*** is used as an auxiliary calibration model input, the correction model is referred to as *C*(*PM*_*s.x*_,***v***,Δ***v***;***p***).

Evaluation of differentials requires storing only one additional set of measurements, namely, one previous sensor reading. However, their capture the local temporal changes in environmental conditions, potentially aiding in the prediction of future alterations. Additionally, they may enhance understanding of the dynamics of explicit or implicit factors influencing LCS operation.

During ANN model identification, the employment of differentials results in extension of the training dataset by adding differential vectors Δ***v***^(*b.j*)^, *j* = 1, …, *N*_*b*_, corresponding to all original training samples. Because the samples are allocated sequentially in time, i.e., the sample with index *j* has been acquired *dt* later than the sample with index *j* – 1, we simply have Δ***v***^(*b.j*)^ = [***v***^(*b.j*)^ – ***v***^(*b.j*–1)^]/*dt*, *j* = 1, …, *N*_*b*_.

**Table 2:** Auxiliary inputs of the correction model expressed as differentials.

Environmental parameter	Differential
External temperature	$\Delta T_{o}^{{}}={{\left[ {{T_o}(0) - T_{o}^{{}}( - dt)} \right]} \mathord{\left/ {\vphantom {{\left[ {{T_o}(0) - T_{o}^{{}}( - dt)} \right]} {dt}}} \right. \kern-0pt} {dt}}$
Internal temperature	$\Delta T_{i}^{{}}={{\left[ {{T_i}(0) - T_{i}^{{}}( - dt)} \right]} \mathord{\left/ {\vphantom {{\left[ {{T_i}(0) - T_{i}^{{}}( - dt)} \right]} {dt}}} \right. \kern-0pt} {dt}}$
External humidity	$\Delta H_{o}^{{}}={{\left[ {{H_o}(0) - H_{o}^{{}}( - dt)} \right]} \mathord{\left/ {\vphantom {{\left[ {{H_o}(0) - H_{o}^{{}}( - dt)} \right]} {dt}}} \right. \kern-0pt} {dt}}$
Internal humidity	$\Delta H_{i}^{{}}={{\left[ {{H_i}(0) - H_{i}^{{}}( - dt)} \right]} \mathord{\left/ {\vphantom {{\left[ {{H_i}(0) - H_{i}^{{}}( - dt)} \right]} {dt}}} \right. \kern-0pt} {dt}}$
Atmospheric pressure	$\Delta P={{\left[ {P(0) - P( - dt)} \right]} \mathord{\left/ {\vphantom {{\left[ {P(0) - P( - dt)} \right]} {dt}}} \right. \kern-0pt} {dt}}$
Aggregated parameters	$\Delta {\mathbf{v}}={\left[ {\Delta {T_o}\;\Delta {T_i}\;\Delta {H_o}\;\Delta {H_i}\;\Delta P} \right]^T}$

Another type of auxiliary calibration inputs are short time series of previously collected PM_*x*_ measurements provided by LCS. More specifically, we consider vectors of the form

14 $${{\boldsymbol{w}}_K}={\left[ {P{M_{s.x}}( - Kdt)\;P{M_{s.x}}( - (K - 1)dt)\;\ldots\;P{M_{s.x}}( - 2dt)\;P{M_{s.x}}( - dt)} \right]^T}$$

where *K* refers to the time series length, and *dt* denotes the inter-measurement time interval. Time series is often handled using recurrent neural networks (RNN)^69^. Nevertheless, here, fixed-length series will be considered, which makes feedforward networks a sufficient and simpler tool. It can also be noted that using *K* = 1, is equivalent to employing a differential of the primary reading PM_*x*_. If the time series vector ***w***_*K*_ is used as an auxiliary calibration model input, the correction model may be referred to as *C*(*PM*_*s.x*_,***v***,***w***_*K*_;***p***), or *C*(*PM*_*s.x*_,***v***,Δ***v***,***w***_*K*_;***p***) if both ***w***_*K*_ and differentials are utilized as well.

The reason for integrating the time series data is to enable the calibration model to understand the typical temporal variations in the LCS readings, including its dependency on environmental conditions. This may enhance the reliability of predicting the values of the correction coefficients *A*_*a*_ and *A*_*m*_.

For the purpose of calibration model identification, the training dataset needs to be extended by adding vectors ***w***_*K*_^(*b.j*)^, *j* = 1, …, *N*_*b*_, corresponding to all *N*_*b*_ original samples. Because the sample indices are in correspondence with the timestamps of respective measurements, i.e., sample with index *j* – 1 was acquired *dt* earlier than sample with index *j*, sample with index *j* – 2 was taken *dt* earlier than that with sample *j* – 1, and so on, the vector ***w***_*K*_^(*b.j*)^ takes the form of ***w***_*K*_^(*b.j*)^ = [*PM*_*s.x*_^(*b.j*–*K*)^ *PM*_*s.x*_^(*b.j*–*K*+1)^ … *PM*_*s.x*_^(*b.j*–2)^ *PM*_*s.x*_^(*b.j*–1)^]^*T*^.

### Calibration procedure

**Fig. 6:**
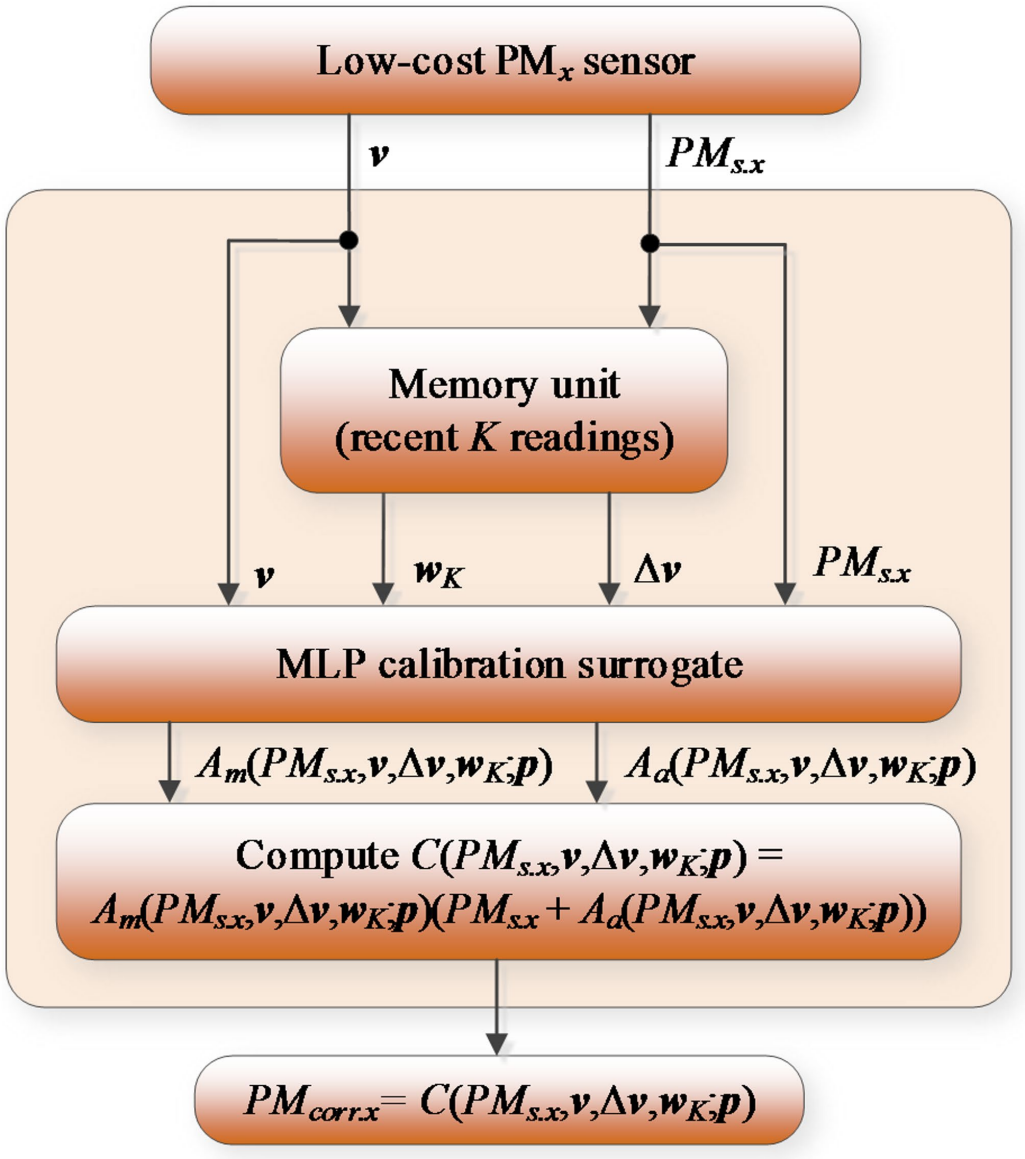
Operational flow of the developed calibration procedure. The input includes the following readings: PM_*x*_ and environmental parameters (vector ***v***). The MLP model assesses correction coefficients, which permit obtaining the calibrated output *PM*_*corr.x*_.

Figure 6 illustrates the PM_*x*_ measurement process utilizing the corrected LCS readings. The initial step involves preparing the calibration inputs, which, if utilizing differentials and time series, necessitates accessing prior sensor readings stored in a memory unit. Subsequently, in the second step, the MLP calibration model generates predicted correction coefficients, which are subsequently employed to calculate the corrected outputs of the LCS. It should be mentioned that the work^70^ discusses an alternative calibration procedure employing ANN and time series alignment, where the calibrated model output is produced directly by the surrogate. In contrast, the technique proposed here is primarily based on a combination of multiplicative and additive corrections with an optimizable control factor, which enhances the calibration process flexibility.

## Results and discussion

Here, we focus on validating the introduced calibration strategy, applied to the mobile measurement platform. The field calibration process utilizes reference data collected by the equipment of governmental stations outlined in Sect. Collecting Reference Data, along with the LCS data obtained from portable platforms situated near the respective reference stations. Section Training and Testing Data explores the reference and LCS data, including their partitioning into training and testing sets. The experimental setup is explained in Sect. Results, detailing the employed scenarios. Key points of investigation include the effects of affine versus additive-only correction, MLP architecture optimization, and the significance of different calibration inputs, particularly differentials and the sensor’s time series. Numerical results are compiled within this section as well. Section Discussion analyses the calibration process performance and offers an in-depth discussion of the paper’s findings.

### Training and testing data

**Fig. 7:**
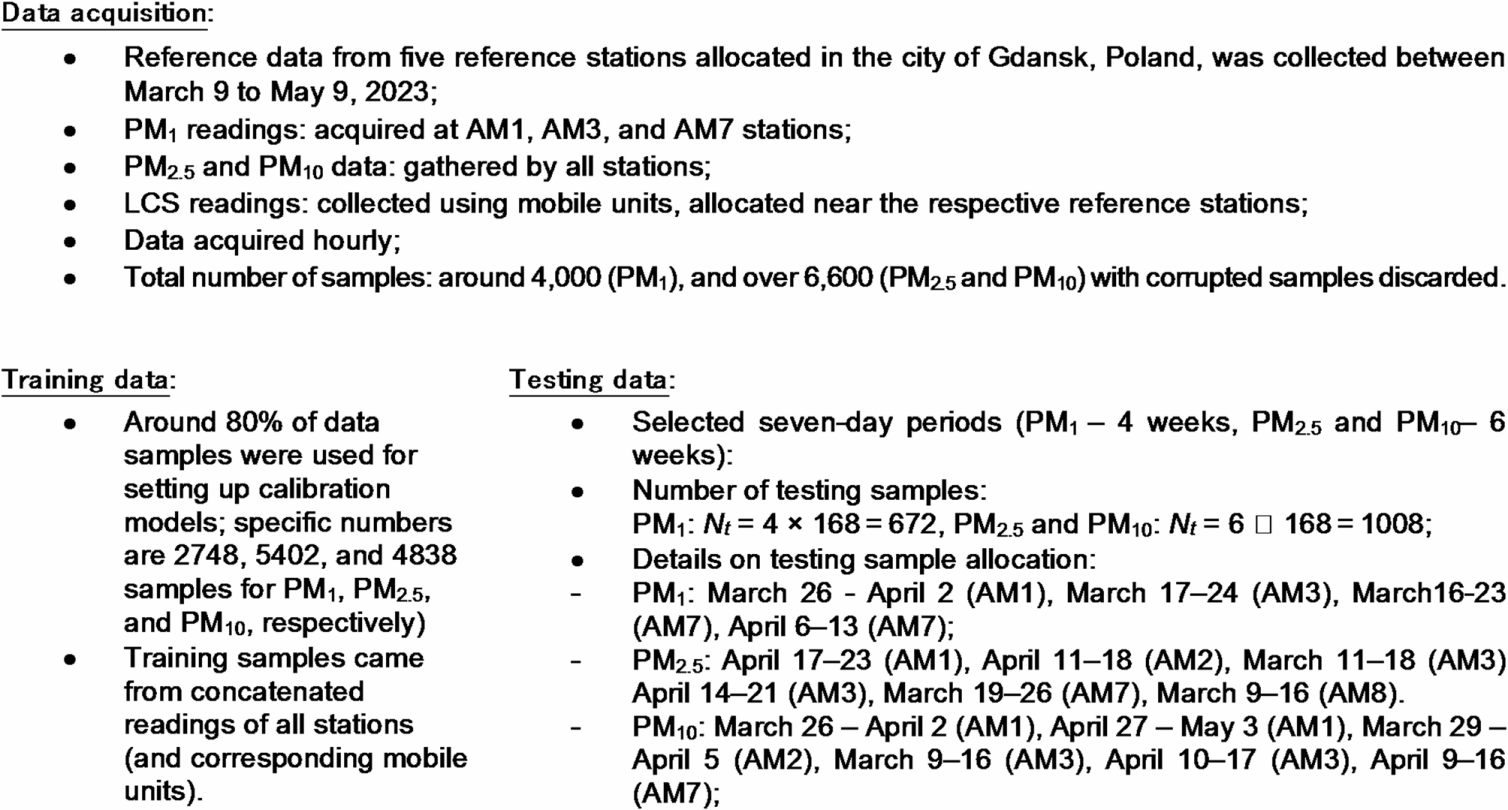
Training and testing data acquired to validate the proposed calibration procedure.

**Fig. 8:**
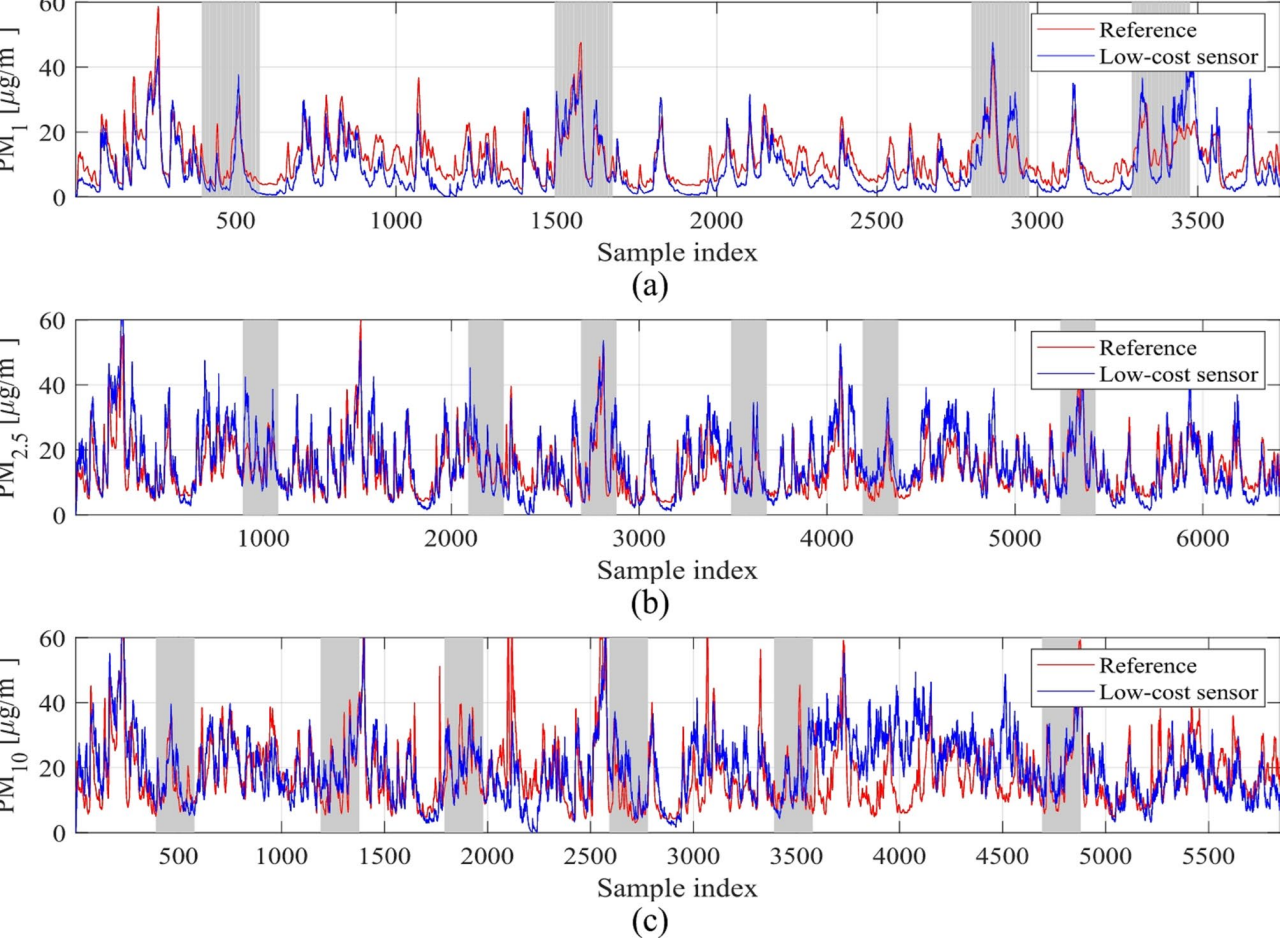
LCS and reference data used for sensor calibration. Grey periods indicate testing data, other samples are utilized to train correction model: (**a**) PM_1_, (**b**) PM_2.5_, (**c**) PM_10_.

The calibration methodology introduced in Sect. ML-based Sensor Correction was demonstrated using the mobile hardware units of Sect. 2, along with reference data acquired at five stationary monitoring stations located in Gdansk, Poland, as detailed in Sect. 3 of this paper. The reference and LCS data were gathered over a nearly two-month period, spanning from March to May 2023. The developed measurement units were positioned in the vicinity of their respective reference stations. Measurements of PM_*x*_ and environmental parameters were recorded hourly. The entire dataset was partitioned into training and testing subsets in a 5:1 ratio. Testing data was organized into seven-day intervals, spanning four and six weeks for PM_1_ and both PM_2.5_ and PM_10_, respectively. Detailed information regarding data acquisition and the composition of the training and testing sets can be found in Fig. 7. Meanwhile, Fig. 8 illustrates the combined reference and LCS measurements for all five stations, with testing periods highlighted in grey. It should be mentioned that our main objective in selecting the allocation of the testing data was to ensure that it represents several consecutive periods (rather than individual samples randomly picked up from the data pool). On the one hand, this makes the calibration problem considerably more challenging. On the other hand, the distribution of testing periods corresponds to different PM levels (low, medium, and high), thereby providing a dependable representation of the sensor’s actual working conditions.

It should also be mentioned that the ranges of environmental parameters throughout the data acquisition periods were quite broad. In particular, the recorded internal temperature *T*_*i*_ changed between 7 C° and 35 C°, the range of the external temperature *T*_*o*_ was − 2 C° to 25 C°. The ranges of internal and external humidity *H*_*i*_ and *H*_*o*_ were 9–49% and 30–82%, respectively, whereas the range of atmospheric pressure *P* was from 974 hPa to 1030 hPa.

These numbers cover typical environmental conditions that might be encountered in urban areas, especially in central and northern parts of Europe. As mentioned earlier, the internal temperature is higher than the external one as a result of heating by the electronic devices installed in the measurement unit. For the same reason, the internal humidity is lower than the external one.

### Results

**Fig. 9:**
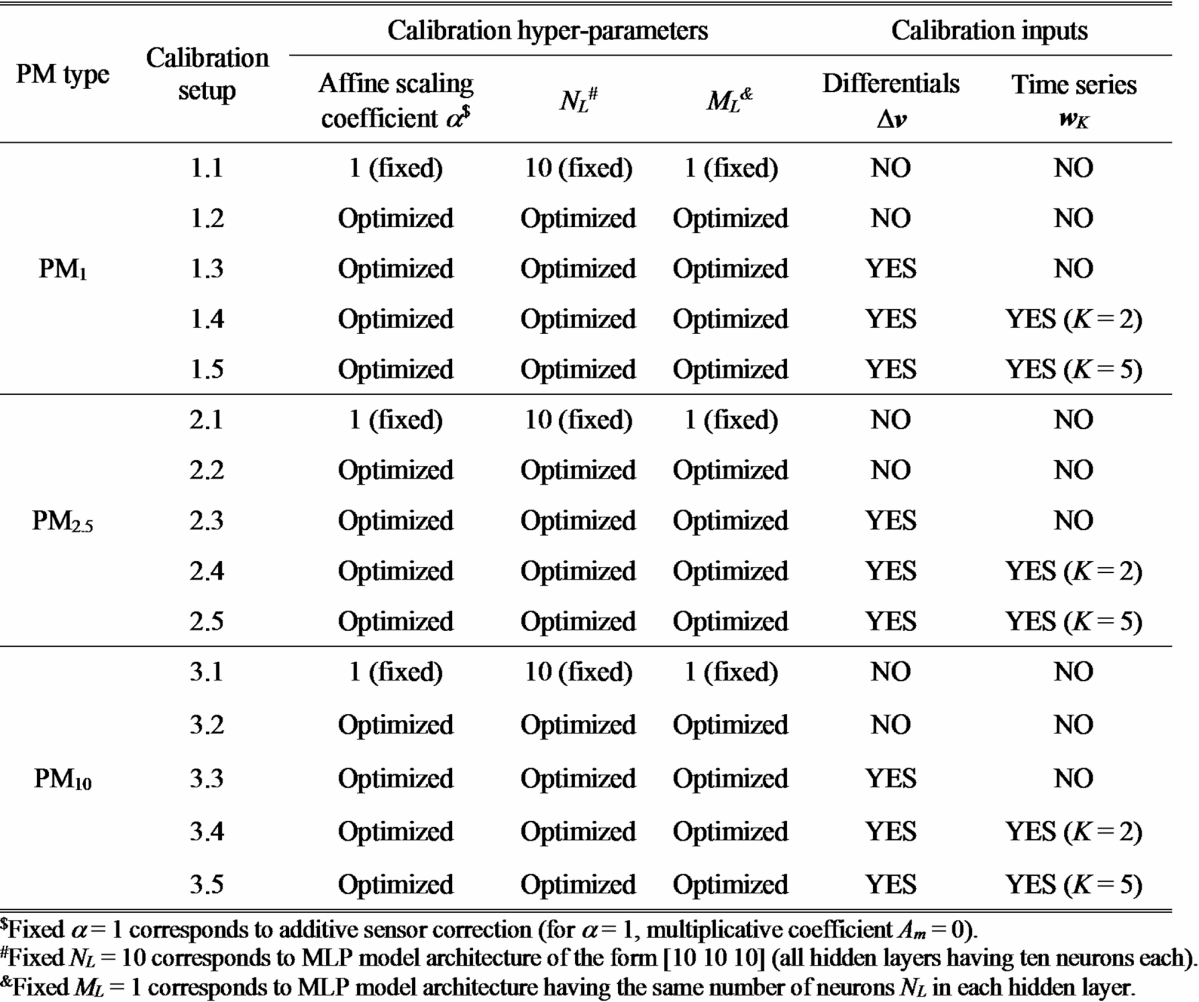
Considered input configurations of calibration models.

In this section we compile the results obtained for calibration of the PM_*x*_ sensors installed on the mobile hardware units of Sect. Sensor Hardware and Software. The results have been gathered for all categories of pollutants, i.e., PM_10_, PM_2.5_, and PM_1_. We considered numerous scenarios presented in Fig. 9. The first two scenarios address different levels of hyper-parameter optimization: (i) fixed *α* = 1 (corresponding to purely additive correction), and (ii) optimizable *α* and optimizable neural network architecture (i.e., adjusting the complete hyper-parameter vector ***H*** = [*N*_*L*_ *M*_*L*_ *α*]^*T*^). The remaining scenarios correspond to fully optimized vector ***H***, and different setups concerning calibration inputs. We consider the basic setup with vector ***v*** comprising environmental parameters as the only input, usage of differentials Δ***v***, and utilization of time series of previous PM_*x*_ samples ***w***_*K*_ acquired by LCS The calibration scenarios referring to PM_1_, PM_2.5_, and PM_10_ were labeled as 1.*k*, 2.*k*, and 3.*k*, *k* = 1, 2, …, 5, respectively. In each configuration, model identification and optimization were conducted fifty times, with the selection of the best setup based on the achieved loss function value to determine the ultimate MLP model. Multiple iterations were required due to the random internal split of testing and training data employed by the training algorithm.

Table 3 presents a summary of the results obtained for all examined calibration scenarios and types of PM. The table includes the coefficient of determination between the reference and corrected LCS data, as well as the root mean squared error (RMSE), calculated for the testing and testing data sets. It is essential to note that our primary interest lies in the sensor’s performance over the testing data, as it reflects the generalization capability of the calibration process and is crucial for assessing the practical utility of the corrected LCS. Definitions of the coefficient of determination and RMSE can be found in Sect. Calibration Task (Fig. 3(d)).

**Table 3:** Performance of the introduced sensor calibration process.

PM type	Calibration setup	Training data		Testing data		Calibration model architecture	
		Coefficient of determination *r*^2^	RMSE [µg/m^3^]	Coefficient of determination *r*^2^	RMSE [µg/m^3^]	*α*	MLP architecture
PM_1_	1.1	0.86	2.57	0.817	3.64	1	[10 10 10]
	1.2	0.91	2.11	0.863	3.13	0.8	[40 20 10]
	1.3	0.93	1.84	0.867	3.10	0.6	[30 15 8]
	1.4	0.90	2.20	0.889	2.95	0.7	[8 15 30]
	1.5	0.92	1.95	0.888	2.97	0.6	[15 15 15]
PM_2.5_	2.1	0.88	2.61	0.814	4.69	1	[10 10 10]
	2.2	0.92	2.10	0.860	4.08	0.7	[6 12 24]
	2.3	0.91	2.25	0.862	4.05	1.0	[20 10 5]
	2.4	0.96	1.51	0.864	4.01	0.8	[10 20 40]
	2.5	0.86	2.77	0.870	3.93	0.7	[5 10 20]
PM_10_	3.1	0.70	5.73	0.721	5.37	1	[10 10 10]
	3.2	0.87	3.83	0.762	4.92	0.8	[30 15 8]
	3.3	0.85	4.12	0.766	4.92	0.9	[24 12 6]
	3.4	0.86	3.84	0.758	5.01	0.7	[24 12 6]
	3.5	0.86	3.91	0.766	4.93	0.6	[20 20 20]

**Fig. 10:**
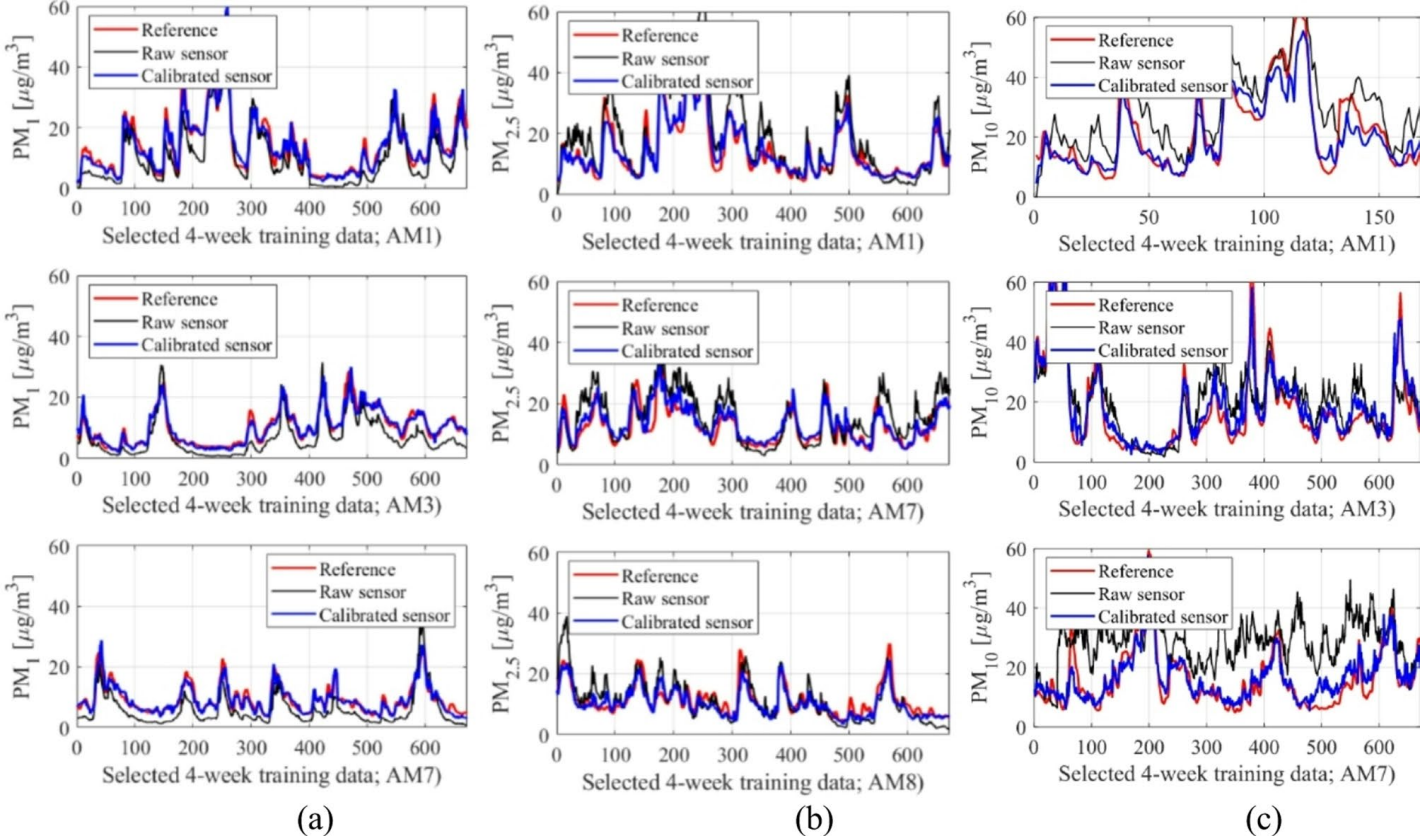
Training data: selected intervals (uncorrected LCS and reference data marked black and red, respectively, corrected readings indicated using blue): (**a**) PM_1_, (**b**) PM_2.5_, (**c**) PM_10_.

**Fig. 11:**
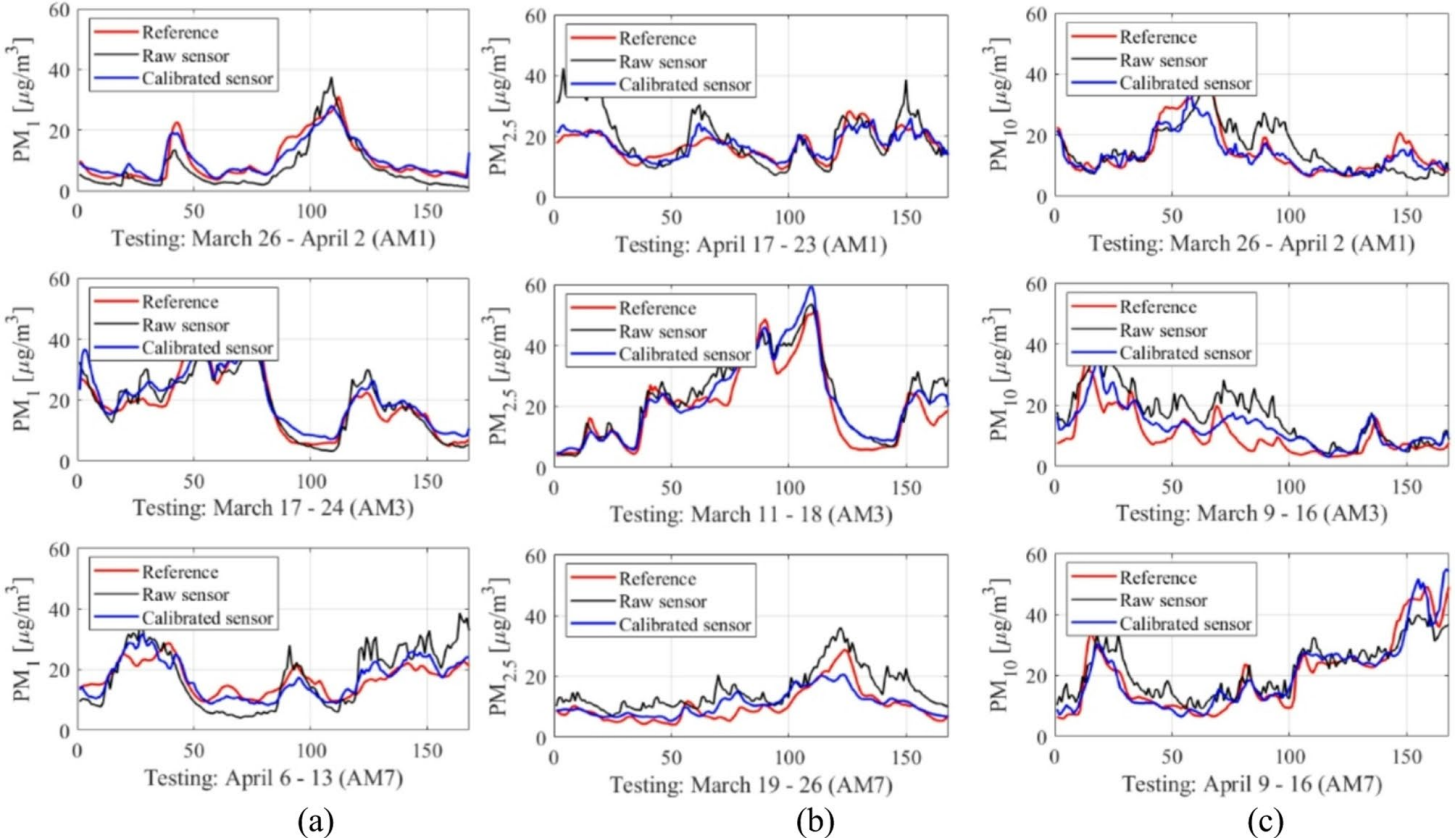
Testing data: selected intervals (uncorrected LCS and reference data marked black and red, respectively, corrected readings indicated using blue): (**a**) PM_1_, (**b**) PM_2.5_, (**c**) PM_10_.

**Fig. 12:**
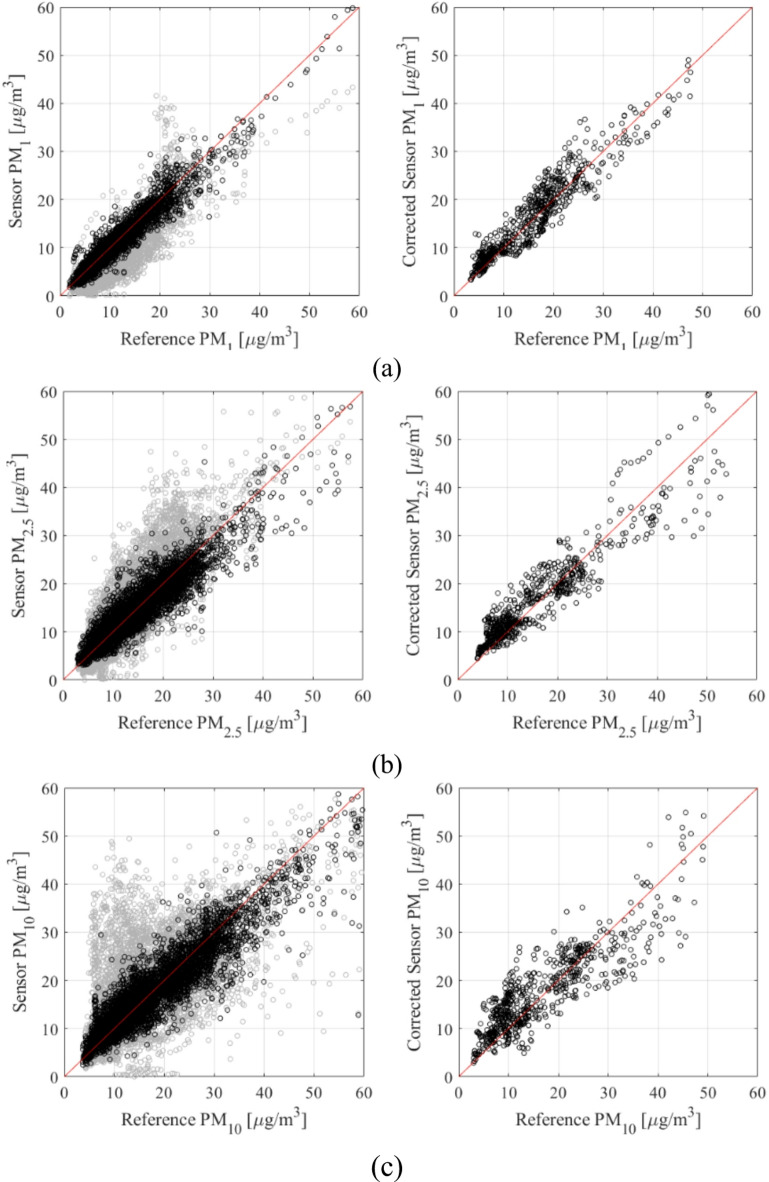
(left) Scatter plots of the training data (corrected samples are marked black, uncorrected data are shown using grey color), (right) scatter plots of the testing data: (**a**) PM_1_, (**b**) PM_2.5_, (**c**) PM_10_.

Reference and calibrated LCS data are presented in Figs. 10 and 11, respectively. Figure 12 displays the PM_*x*_ samples for selected training intervals, Fig. 11 shows the testing data, and Fig. 12 presents the scatter plots. The most comprehensive setup of each PM type (configurations 1.5, 2.5, and 3.5) is illustrated, which includes full hyper-parameter optimization and incorporates environmental variable differentials, as well as sensor time series, as additional calibration inputs.

### Discussion

The performance of the developed calibration methodology was verified by assessing the accuracy of the calibrated LCS in terms of its correlation with the reference data and typical error levels. These metrics are crucial for determining the practical utility of the corrected sensor for monitoring particulate matter pollution. Additionally, we investigated the significance and impact of various constituent parts of the calibration procedure, such as the utilization of affine response scaling, hyper-parameter optimization of the MLP model, and the incorporation of supplementary inputs (temporal changes and series of previous PM_*x*_ measurements). Observe, that the calibration process poses a significant challenge due to the significant discrepancies between the reference and LCS readings. For instance, the coefficient of determination between the raw sensor measurements and reference data is only 0.40 (for PM_1_), 0.44 (for PM_2.5_), and 0.17 (for PM_10_) Furthermore, the measurements exhibit a wide range, from almost zero to nearly 60 µg/m^3^, while PM_*x*_ values vary significantly over short timeframes.

Despite the challenges mentioned earlier, the proposed calibration methodology demonstrates remarkable reliability. For the most comprehensive configurations 1.5, 2.5, and 3.5 (i.e., optimized hyper-parameter vector ***H***, incorporating supplementary inputs), the coefficient of determination reaches impressive levels of 0.89, 0.87, and 0.77 for PM_1_, PM_2.5_, and PM_10_, respectively. As previously noted, PM_10_ presents the most challenging scenario with an extremely poor correlation (0.17) for raw LCS readings. However, even in this case, our calibration framework was able to produce satisfactory results. These improvements are demonstrated in Figs. 10 and 11, and 12, where visual agreement between reference measurements and corrected LCS data is significantly enhanced in comparison to raw sensor data. Moreover, scatter plots of the corrected sensor exhibit much closer concentration around the identity function in comparison to raw readings. Similar enhancements are observed for RMSE values. For the uncorrected sensor, we have RMSE values of 9.6, 5.6, and 5.3 µg/m^3^ (testing data); analogical data for corrected LCS is 4.9, 3.9, and 3.0 µg/m^3^ for PM_10_, PM_2.5_, and PM_1_, respectively. Analysis of the average relative error indicates values of 29, 22, and 18% for PM_10_, PM_2.5_, and PM_1_, respectively, meaning they are practically acceptable.

In addition to evaluating the overall performance of the calibration methodology, we are interested in analysing the relevance of specific components of the procedure. This includes comparing affine scaling to purely additive correction (i.e., coefficient *α* = 1), assessing the impact of MLP hyper-parameter optimization, and examining the effect of incorporating differentials and time series. As observed in Table 2, varying the coefficient *α* leads to noticeable improvements compared to conventional additive correction. The average increase in the coefficient of determination is approximately 0.04 for PM_1_ (setup 1.2 versus 1.1), and around 0.02 for PM_2.5_ and PM_10_. Visible enhancements have been also obtained by optimizing parameters *N*_*L*_ and *M*_*L*_ of an MLP model, resulting in an increase of the coefficient of determination by 0.03 to 0.04 for all PM categories. Additionally, both factors permit reducing RMSE values, their combined effect amounts to approximately 0.5 µg/m^3^. Further improvements albeit minor ones can be achieved by incorporating environmental parameter differentials as auxiliary calibration inputs (setups 1.3, 2.3, and 3.3 versus 1.2, 2.2, and 3.2). This results in an average improvement of up to 0.01 in the coefficient of determination, and an RMSE reduction of approximately 0.03 µg/m^3^. Utilization of time series data (setups 1.4 and 1.5, 2.4 and 2.5, and 3.4 through 3.5) is also associated with some benefits: an average improvement in the coefficient of determination of 0.01, and an RMSE reduction of approximately 0.1 to 0.15 µg/m^3^. However, increasing the time series length *K* has mixed effects depending on the PM type.

The proposed combination of correction mechanisms, calibration model and its optimization, as well as primary and auxiliary inputs (including supplementary data accounting for local temporal variations of the environmental variables and PM_*x*_ readings) results in a significant improvement in the accuracy of LCS. This is evidenced by the high values of the coefficient of determination, which approach 0.9 (or 0.8 for PM_10_), despite the poor dependability of the raw sensor. Similar improvements are observed for the modelling error, which ranges from three to four µg/m^3^ of RMSE, depending on the PM type. In practical implementation, calibration can be achieved using the built-in computational resources of the portable platform. Alternatively, it can be applied after transmitting the raw sensor readings from the platform (and before making the data available to the end user).

## Conclusion

This study presents an innovative approach to efficiently calibrate low-cost particulate matter sensors in field conditions. Our methodology integrates an artificial neural network surrogate model serving as the main prediction tool determining the coefficients of additive and multiplicative response scaling. The ANN configuration specifically employed is a multi-layer perceptron, whose hidden layers are fully connected. The layers’ sizes are optimized during the model identification process. In addition to environmental parameters assessed by LCS, our calibration inputs include supplementary data. These auxiliary inputs encompass time derivatives of environmental variables and short time series of previous PM_*x*_ samples from the sensor undergoing calibration. Incorporation of these supplementary inputs facilitates learning of typical temporal dependencies between parameters such as temperature, humidity, or atmospheric pressure, and the PM_*x*_ measurements by the MLP model.

The introduced correction scheme was verified using mobile hardware units constructed at Gdansk University of Technology, Poland. The employed equipment included low-cost PM_*x*_ and environmental sensors, as well as electronic circuits designed for carrying out measurements, storing data, and wirelessly transmitting it using the built-in GMS modem. Field calibration of the sensor was performed based on the reference readings gathered by five public monitoring stations located in Gdansk, Poland. Corresponding LCS data was collected using multiple copies of the portable platform located in the vicinity of the reference stations. Extensive experiments were performed involving various configurations of the calibration model. The results obtained demonstrate exceptional reliability of the presented procedure for all three categories of particulate matter: PM_10_, PM_2.5_, and PM_1_. For the most comprehensive calibration setup (optimized MLP hyper-parameters, combined multiplicative/additive scaling, auxiliary inputs), the achieved coefficient of determination between the corrected LCS and reference readings equal 0.89 for PM_1_, 0.87 for PM_2.5_, and 0.77 for PM_10_. These values indicate a remarkable improvement over the uncorrected sensor, which exhibit the coefficient of determination of 0.40, 0.44, and 0.17, respectively. Moreover, the RMSE error levels equal about 3.0, 3.9 and 4.9 µg/m^3^.

Further experiments were conducted to assess the significance and impact of specific constituent parts of the calibration framework. These experiments confirmed the essential nature of these components in achieving top performance in the sensor correction process. In particular, the utilization of combined additive/multiplicative scaling led to an increase of up to 0.04 in the coefficient of determination over additive scaling alone. Optimization of MLP model architecture further contributed up to 0.04 to the average increase in coefficient of determination. While the effects of incorporating supplementary inputs (such as differentials and time series) were less prominent, they were still noticeable. Overall, these components jointly resulted in reducing RMSE from 0.5 to 1.3 µg/m^3^ (e.g., from 3.64 to 2.97 µg/m^3^ for PM_1_).

The future research will also aim at enhancing calibration process reliability even further. One avenue to explore involves considering more sophisticated artificial intelligence methods, particularly convolutional and recurrent neural networks, as potential calibration models. Additionally, other options include developing global correction mechanisms aimed at reducing discrepancies between LCS and reference data at the level of complete datasets, before applying specific correction mechanisms at the level of individual sensor measurements.

## Data Availability

Data availability: The datasets generated during and/or analysed during the current study are available from the corresponding author on reasonable request. Contact person: anna.dabrowska@pg.edu.pl.
